# Effect of methylation on genome mutability in cattle

**DOI:** 10.1186/s12711-026-01051-y

**Published:** 2026-06-17

**Authors:** Corentin Fouéré, Valentin Costes, Chris Hozé, Florian Besnard, Gabriel Costa Monteiro Moreira, Sébastien Fritz, Hélène Kiefer, Marie-Pierre Sanchez, Didier Boichard, Mekki Boussaha

**Affiliations:** 1 Eliance, 75012 Paris, France; 2 Université Paris-Saclay, INRAE, AgroParisTech, GABI, 78350 Jouy-en-Josas, France; 3 INRAE, BREED, Université Paris-Saclay, 78350 Jouy‑en‑Josas, France; 4 Ecole Nationale Vétérinaire d’Alfort, 94700 BreedMaisons‑Alfort, France

## Abstract

**Background:**

Germline de novo mutations (DNMs) are rare events in mammals, typically occurring only a few dozen times per generation. These mutations are not entirely random; several factors are known to influence their rate, including DNA methylation. In this study, we leveraged the unique population structure of cattle with a few ancestors having a large contribution to the current gene pool, along with comprehensive genomic resources, to investigate mutational processes. We applied two complementary approaches: (1) identifying DNMs accumulated over generations in family trio segments (from identical-by-descent segments between descendants and direct ascendants spanning several generations) in Holstein and Montbéliarde breeds, and (2) detecting rare bi-allelic substitution variants (Minor Allele Frequency < 0.001) from a large panel of sequenced Holstein animals.

**Results:**

Overall, transitions were over-represented compared to transversions for both DNMs and rare substitutions. Considering the nucleotide context, a notable enrichment of C > T substitutions was observed within CpG sites (CpG > TpG). This enrichment was particularly pronounced in low CpG density regions and positively correlated with local methylation levels in both gametes and several somatic tissues. Additionally, several transposable elements exhibited higher mutation rates relative to the rest of the genome, particularly young SINE and LINE elements.

**Conclusions:**

Together, these results provide insights into the mutational landscape in cattle and reinforce observations made in other mammalian species.

**Supplementary Information:**

The online version contains supplementary material available at 10.1186/s12711-026-01051-y.

## Background

In animals, the transmission of genetic material across generations is extremely accurate. Among the billions of nucleotides that make up an individual's DNA sequence, only a few dozen correspond to mutations absent in the parental genetic material—referred to as de novo mutations (DNMs). Such DNMs are the original source of genetic variation [1], influence the pace of genome evolution [1], and can lead to recessive genetic defects [2, 3].

The germline mutation rate (GMR) per generation has been investigated in a wide range of species. Across 36 mammalian species, the average GMR is approximately 7.97 × 10^−9^ persite [4]. In cattle, based on a large cohort of 131 trios, Harland et *al.* (2018) estimated the GMR at 1.2 × 10^−8^ per site [5], a value comparable to estimates in humans [6, 7]. Several factors are known to influence DNM rate. In particular, cytosine methylation has been reported to increase the prevalence of DNMs in mammals by a factor of 10–50 [8], mainly due to spontaneous deamination of 5-methylcytosine (5mC) to thymine. Other factors include parental origin bias [4]: DNMs originate more frequently from the father than from the mother, with a reported male-to-female ratio of 1.8:1 in cattle [5]. Assisted reproductive technologies, such as in vitro fertilization (IVF), have also been reported to increase mutation rates. Specifically, de novo structural variants appear five times more frequently in embryos generated by IVF, primarily during the one- and 2-cell stages [9].

In livestock species, the decrease in sequencing costs along with international initiatives have led to the sequencing of hundreds to thousands of individuals [10, 11]. In parallel, the widespread use of artificial insemination has led to the disproportionate dissemination of genetic material from a limited number of sires. For example, within the French Montbéliarde breed, eight bulls born between 1971 and 1996 account for nearly 58% of the genetic heritage of current active females [12]. This unique population structure, combined with the availability of genomic sequence data from both ancestors and contemporary animals, provides an opportunity to detect DNMs accumulated over successive generations.

In this study, we leverage a large number of whole-genome sequences and founder-like effect of major dairy cattle breeds to investigate mutational patterns in French Holstein and Montbéliarde populations. We used two complementary strategies: (1) identification of rare bi-allelic substitution variants across a large panel of sequenced animals, and (2) detection of DNMs within multigenerational family trios using identical-by-descent (IBD) segments between descendants and direct ancestors (ascendants in the direct lineage, separated from the descendant by multiple generations). In other words, for the second strategy, we examined shared genomic regions in which both haplotypes of a modern animal were inferred to be IBD with those of sequenced ancestors. We then further explored bi-allelic substitutions, focusing on their potential enrichment in relation to genomic features such as cytosine methylation.

## Methods

### Animals

This study used whole-genome sequence data from a total of 507 animals, comprising 369 Holstein (Hol) and 138 Montbéliarde (Mon) individuals, each with a genome coverage of at least 10x (Mean_Hol_ = 16.2x; Mean_Mon_ = 16.8x). These sequences were obtained from previous projects conducted by our research team. The sequenced animals were separated into two distinct groups based on birth year: ancestors, born prior to 2004 in Holstein (N_ascendants Hol_ = 81) and Montbéliarde (N_ascendants Mon_ = 49); and progeny, born after 2010 for the Holstein breed (N_descendants Hol_ = 288) and after 2016 for the Montbéliarde breed (N_descendants Mon_ = 89). To complete this dataset, we used Holstein sequences from the 1000 Bull Genomes Project [10], which include sires from external origins not sequenced by our team but influential in the Holstein pedigree. This expanded the number of Holstein sequences analysed to 1,148.

### Variant calling and VCF creation

Whole genome sequencing (WGS) of the 369 Holstein and 138 Montbéliarde animals was performed at the INRAE Get-PlaGe platform (http://genomique.genotoul.fr/). Paired-end libraries with insert sizes ranging from 300 to 500 bp were constructed using either the NEXTflex PCR-Free DNA Sequencing Kit (Biooscientific) or the TruSeq Nano DNA Library Prep Kit (Illumina). Libraries were subsequently sequenced on Illumina machines (HiSeq2000, Hiseq3000, or NovaSeq 6000) producing either 2 × 100-bp or 2 × 150-bp read lengths. Paired-end reads were subsequently aligned to the bovine ARS-UCD1.2 reference genome sequence using the BWA mem algorithm (BWA-v0.7.17) [13]. SNPs and small InDels were called using the GATK-HaplotypeCaller software (v3.8 and v4.4) to produce GVCF files for each sample. Subsequently, GenotypeGVCFs was used to genotype GVCFs using the parameter “–allow-old-rms-mapping-quality-annotation-data” which enables genotyping of GVCFs generated with older versions of GATK and converts the GVCF files into VCF format [14] following the GATK best practice guidelines, as previously described [15]. Only variants located on autosomes were considered in subsequent analysis.

### Identification and validation of identical-by-descent (IBD) segments

**Fig. 1:**
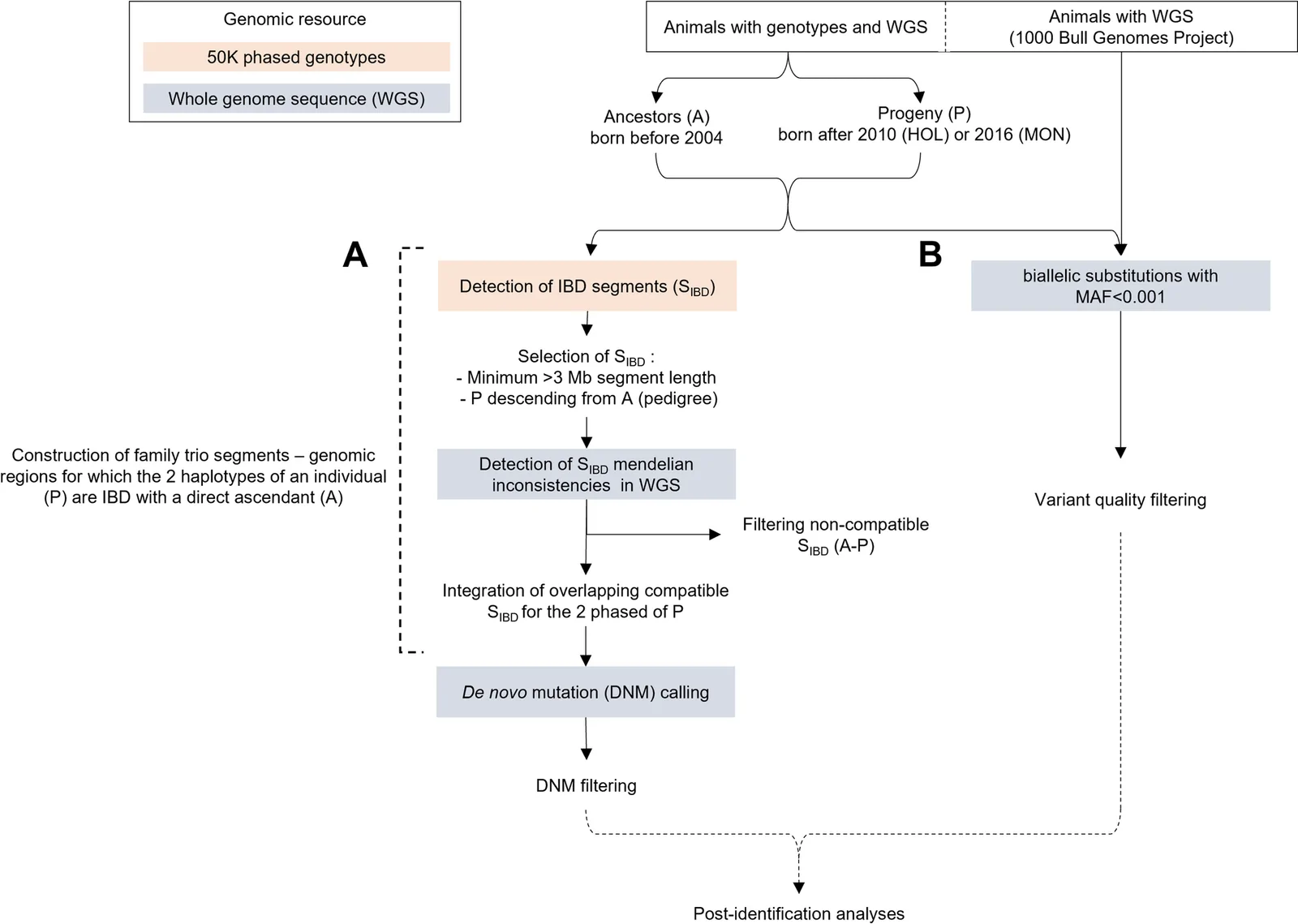
Overview of the methods for selection of mutations **a** Schematic of the family trio segments. Animals with genotypes and whole genome sequence are divided in two groups: ancestors (A) and progeny (P). Phased haplotypes shared between A and P are identified using 50 K phased genotypes. The haplotypes are then tested at the sequence level, with those exhibiting a high rate of Mendelian error between A and P being discarded. The remaining segments are assembled to create family trio segments (a portion on which the two phases of P are assigned to direct ascendant haplotypes with available sequence data), which are then searched for de novo mutations. **b** Rare bi-allelic substitutions are obtained a large panel of sequenced animals. Mutations obtained from family trios that span multiple generations and rare mutations are then further explored for mutational patterns

The generation of 50 K SNP genotypes was similar to that previously reported by Jourdain et al. [16]. Briefly, genotypes were obtained using several SNP arrays (low-density, custom-LD, BovineSNP50, EuroGMD and high-density). As part of the French routine evaluation, the raw genotypes were then imputed and phased for 53,469 autosomal SNPs using Fimpute3 [17]. Due to the large number of their genotyped progeny, the phases of the sequenced animals were assumed to be known without error. Phased 50 K genotypes of all sequenced animals were used to identify genomic segments shared between a progeny’s haplotype and that of an ancestor. Candidate segments were defined as putative IBD regions and were required to be at least 3 Mb in length. These candidate segments were subsequently tested for Mendelian inconsistencies using whole-genome sequence data. Mendelian inconsistencies were defined as positions where the progeny and the direct ancestor had opposing homozygous genotypes for a given variant (*e.g.,* progeny = AA and ancestor = CC). Only sites with genotype quality (GQ) > 40 in each of the two individuals were considered. To address the uncertainty surrounding the segment boundaries, a 250 kb region was removed from each end of every candidate segment. Segments were retained if the remaining core region had less than three Mendelian inconsistencies per 10 Mb on average. A final set of non-overlapping segments was selected for each haplotype in the progeny. In case of overlap (*i.e.*, when a common genomic region was attributed to two ancestors), the longest segment was selected. Subsequently, the two haplotypes of the progeny were merged to establish family trio segments—consisting of one progeny and its direct ascendants: a genomic region where each of the two haplotypes in the progeny is shared with one direct ascendant and is considered to be inherited from that direct ascendant (*i.e.*, considered IBD). For each segment, the number of generations that separates the progeny from the direct ascendant was estimated as the average number of generations between them, based on the pedigree information. The main steps of the construction of family trio segments are illustrated in Fig. 1a.

### Detection and filtering of de novo mutations

DNMs were detected only on autosomes. The main steps are illustrated in Additional file 3 Fig. S1. Candidate DNMs were firstdetected on each family trio segment as heterozygous variants in the progeny (0/1) for which both ancestors possess the identical homozygous genotype (0/0 or 1/1). Additional filtering for the considered variant included (1–4):

1. Keeping high-quality region variants based on GATK metrics (https://gatk.broadinstitute.org/hc/en-us/articles/30331989211419--Tool-Documentation-Index) (*i.e.*, those with MQ > 40 and QD > 2 and FS < 20 and -2 < MQRankSum < 4 and -3 < ReadPosRankSum < 3 and SOR < 3; [18]);
2. For each individual in the trio, a read depth ranging from 10 reads to twice the mean sequencing depth (mean(DP)) [18, 19];
3. A GQ > 40 for each individual [18];
4. An allelic balance between 0.3 and 0.7 for the candidate DNM in the progeny [18].

We applied a further set of filters to the candidate DNMs and their family trio segments. First, we trimmed a 0.8 Mb portion from both ends of the segments, as we observed an abnormally high density of candidate DNMs in these regions (candidate DNMs are expected to occur at random locations on the segments). This enrichment may be due to inaccuracies in the segment boundaries. Secondly, we discarded the refined segments that presented an average of > 1 candidate DNM per Mb. Finally, candidate DNMs were retained if no more than two were observed within the same Mb of a given segment.

Lastly, we assess the local sequence quality. Each candidate DNM was evaluated using the raw VCF. The number of variants within a ± 75 bp window of the candidate DNM (~ length of one short read) was computed for the progeny and the two direct ascendants. Candidate DNMs were retained when the difference between the number of variants in the ± 75 bp area of the progeny and the total count of variants from the two ancestors in this same area was ≤ 1 (to account for candidate DNM). Candidates with higher DNM values were excluded as they were probably due to alignment problems, including poor quality local alignment.

Several DNMs were identified across multiple family trio segments but corresponded to the same mutation at the same genomic position. Likely they correspond to DNMs that occurred in a common path between an ancestor and two descendants. To avoid inflating mutation counts and introducing bias in downstream analyses, each such DNM was counted only once in subsequent analyses (*i.e.*, trinucleotide context, enrichment analyses and methylation-level analysis).

### Estimation of callable genome

The callable genome proportion was estimated for each trio segment (trimmed by the 0.8 Mb portion on both sides) for which the GVCFs files were available for the progeny and both direct ascendants. The callable genome proportion of a trio segment was defined as the set of common sites in the three GVCFs (including both polymorphic and non-polymorphic sites), provided that the filters (2) and (3) (see “Detection and filtering of de novo mutations” section) were respected. The callable genome rate was only computed for the Montbéliarde breed because the GVCFs of multiple Holstein animals were not available.

### Estimation of the mutation rate

In the Montbéliarde breed, we used the callable genome rate to determine the mutation rate. For a given generation, we divided the number of DNMs by the sum of the callable genome length of all corresponding segments. When multiple paths were possible between a descendant and its ascendants based on pedigree information, the considered generation separating the individuals was the average of all possible paths. When the mutation was observed in multiple descendant-ascendants trios, the generation considered was the average across the different trios, and the segment length was the average of all the segments of the considered trios. We then performed a weighted linear regression of the observed mutation rate in each generation class, using the callable genome length of the corresponding class as the weighting factor. The slope of this regression reflects the mutation rate per generation.

### Identification of rare substitutions variants

Aside from DNMs, we used the VCFs of the 1000 Bull Genomes Project to select rare bi-allelic substitution variants (referred to as rare substitutions further in the manuscript). First, we constructed a genomic relationship matrix from the genotypes (53,469 autosomal SNPs) using PLINK [20] to verify that closely related individuals represented only a minority fraction of the selected animals. We retained only variants with a minor allele frequency (MAF) below 0.001 within the breed (*i.e.,* allele frequency (AF) below 0.001 or above 0.999) and located in high-quality regions (defined using the same filters as in step (1) of the “Detection and filtering of de novo mutations” section). At this frequency, one or two copies of the rarest allele were observed in the Holstein breed. Although animals homozygous for the rare variants may occur because of recent inbreeding, we decided to exclude these variants for which both copies of the rarest allele were carried by the same individual. Consequently, we retained only variants for which one or two heterozygous animals were observed. We then computed their AF in all breeds of the 1000 Bull Genomes Project and only kept those with a MAF < 0.001. As the number of sequenced animals in Montbéliarde was less than 500, a MAF < 0.001 was not achievable, and thus, we only conducted the analyses for the Holstein breed. The direction of substitution was defined as the change from the frequent allele to the rare allele.

### Characterization of DNMs and rare substitutions

#### Trinucleotide context

To investigate sequence context biases in mutation patterns, we analyzed the trinucleotide context of each bi-allelic substitution mutation, considering both DNMs and rare substitutions. For each substitution, the trinucleotide context was defined as the mutated base along with its 5’ and 3’ flanking bases, retrieved from the bovine reference genome assembly (ARS-UCD1.2). Because each bi-allelic substitution can be represented in two strand orientations, the same event has 2 trinucleotides versions corresponding to the 5’ > 3’ direction or its complementary 3’ > 5’ reversed (*e.g.*, ACG > ATG and its reverse complement CGT > CAT). The combination of these 2 versions results in 96 unique events (trinucleotide contexts for the 6 possibles substitutions; (4 × 4 × 4 × 3)/2)) [21].

To assess mutation biases, we computed fold enrichment in observed trinucleotide context for the bi-allelic substitutions (DNMs and low frequency SNVs) with the background set corresponding to the hypothesis that all substitutions occur with equal probability within each trinucleotide context. Specifically, for one substitution in a given trinucleotide context, the expected number of substitutions is:

$$\frac{f_{XYZ}}{3}~ \times ~{N}$$

where $f_{XYZ}$ is the observed frequency of trinucleotide *XYZ* in the ARS-UCD1.2 reference assembly, *3* is the number of possible substitutions for the middle nucleotide, and *N* is the total number of observed substitutions. For example, the trinucleotide TTT (and its reverse complement AAA) exhibited a frequency of 6.84% within the reference genome. The central T can mutate to A, C, or G, leading to three possible trinucleotide contexts: TTT > TAT, TTT > TCT and TTT > TGT. Assuming a total of 1000 substitutions observed overall, and under the assumption that mutations are uniformly distributed across all contexts, the expected number of TTT > TAT (and AAA > ATA) substitutions is calculated as follows: (6.84%/3) × 1000 = 22.8.

#### Genomic feature annotations

To annotate mutation sites, we integrated data from several genomic resources. Gene coordinates were obtained from the Ensembl genes database using the Biomart software suite (release 112). CpG islands (CGIs) coordinates were retrieved from the UCSC genome browser (“cpgIslandExt” table) [22] and the CGI landscape was extended to include two flanking regions: (1) CGI shore regions, defined as regions located 0-2 kb from the CGI boundary, and (2) CGI shelf regions, located2-4 kb from the CGI boundary. Genomic regions outside these features were referred to as “open sea”. Transposable Elements (TEs) were annotated using the UCSC genome browser (”RepeatMasker table”) [22], with a focus on LTR, SINE, LINE (class I Retrotransposons), as well as DNA transposons (class II Transposons). Finally, ATAC-seq peaks, which reflect chromatin accessibility, were retrieved from the dataset published by Yuan et al. [23].

#### Enrichment analyses of DNMs

To assess whether DNMs were enriched or depleted in specific genomic features, we compared their distribution to that of a background set consisting of randomly selected genomic positions, while maintaining the proportion of genome covered by all the family trio segments. Specifically, we randomly selected 1,000,000 positions that were distributed across all the analysed segments in proportion to their length, and from this list, we randomly selected a subset equal in size to the number of DNMs detected. These selected positions were annotated in the same way as the DNMs and the enrichment was then quantified using an odds ratio (OR) calculated between the detected DNMs and the background set. This process was repeated 1,000 times to generate a distribution of OR values. The 95% confidence interval (CI) was defined by excluding the 2.5% most extreme ORs on either side of the distribution.

#### Enrichment analyses of rare substitutions

A similar enrichment analysis was conducted for rare substitutions. In this case, the background set consisted of randomly selected genomic positions equal in number to the observed rare substitutions. The 95% CI of the OR was computed as follows: $CI={e}^{(\mathrm{log}(OR)\pm 1.96\times SE(\mathrm{log}(OR)))}$, with the standard error approximated by: ${\mathrm{SE}}\left( {{\mathrm{log}}\left( {{\mathrm{OR}}} \right)} \right) = \sqrt {\frac{1}{n1} + \frac{1}{n2} + \frac{1}{n3} + \frac{1}{n4}}$ with ${n1}$, the sample size annotated, ${n2}$, background set size annotated, the sample size not annotated, and , the background set size not annotated for the corresponding genomic feature.

In addition, we examined the distribution of rare substitutions within ATAC-seq peaks. For every peak, we defined consecutive bins of 200 bp on either side of the peak center (*e.g.*, bin 1: 0–200 bp to the peak, bin 2: 201-400 bp). For each bin *i*, we calculated the frequency of variants aggregated per bin (substitution frequency): $SF_{\text{bin i}} = \frac{{\text{number of variants}_{\text{bin i}} }}{{\text{total length}_{\text{bin i}} }}$. We compared the observed distribution with a set of common substitutions (MAF > 0.2), obtained from the same cohort, and filtered using the same quality criteria outlined in step (1) of the “Detection and filtering of de novo mutations” section. To ensure comparability between both sets, we randomly sampled an equal number of common substitutions as rare ones. For each bin, the 95% CI for ${SF}_{\text{bin i}}$ was derived from the normal approximation using this formula: $SF_{\text{bin i}} \pm 2 \times \sqrt {\frac{{p\left( {1 - p} \right)}}{n}}$; with $p = SF_{\text{bin i}}$ and $n = {total\,length}_{\text{bin i}}$.    

Regarding TE, we computed the relative substitution frequency of various TE elements as ${{Relative\,SF}}_{\text{TE i}}= \frac{{SF}_{\text{TE i}}}{{ SF}_{\text{no TE region}}}$; corresponding to the ratio of the substitution frequency observed in the TE class on the frequency observed in genomic regions not annotated as TE, per substitution type (e.g., C > A, T > C, etc.).    

The last three types of analysis mentioned above (ATAC-SEQ peaks, CpG > TpG frequency around CGIs, and TE element-type enrichment) were only performed for the rare substitutions for which a much larger number of mutations were available.

#### Methylation-level analysis

To investigate the effect of methylation on the mutability of CpG > TpG transitions (*i.e.,* *CG > *TG substitutions), we used whole genome bisulphite sequencing (WGBS) data retrieved from the NCBI Gene Expression Omnibus (GEO). These data included tissues involved in the transmission of genetic material (immature oocyte, oocyte and sperm (GSE121758; [24])), as well as early embryonic stages (2-cell, 4-cell, 8-cell, and 16-cell embryo), all from accession GSE121758) [24]. In addition, somatic tissues unrelated to the process of cell multiplication (blood, liver, and adipose tissue) were obtained from accession GSE147087 [25]. The genomic positions of WGBS samples retrieved from the accession number GSE121758 [24], originally mapped to UMD3.1.1, were converted to ARS-UCD1.2 using the R package”rtracklayer” and the UCSC LiftOver file”bosTau8ToBosTau9.over.chain” (https://hgdownload.soe.ucsc.edu/goldenPath/bosTau8/liftOver/bosTau8ToBosTau9.over.chain.gz). To ensure accurate methylation levels, all positions within the WGBS files that overlapped with known genetic variants (including low-frequency SNVs under study) were excluded from the analyses to avoid technical bias. Depending on the type of tissue, between one and five WGBS samples were available.

For each tissue, we combined the WGBS samples and computed methylation levels in 1000 bp genomic bins. The methylation level of each bin was calculated as the total number of methylated cytosine reads within the bin divided by the total number of cytosine (methylated and unmethylated) reads in the bin. All bins containing fewer than five total cytosine reads were excluded from further analyses. Each CpG site within the remaining bins was then assigned the methylation level of the corresponding bin. For each tissue, similar to the methodology of Kohailan et al. [26], we quantified the number of methylated (> 50%) and unmethylated (≤ 50%) CpGs that did not overlap with a known genetic variant (constituting the background set). Additionally, we assessed the number of methylated (> 50%) and unmethylated (≤ 50%) CpGs that exhibit CpG > TpG substitution for both DNMs and rare substitutions. The 50% threshold was chosen based on the typically bimodal distribution of methylation in most cell types [27]. Subsequently, we computed odds ratios (ORs) and performed a two-sided binomial test to determine if the methylation levels for the CpG > TpG substitutions DNMs differed from those of all CpGs. For rare CpG > TpG substitutions, we computed the 95% CI of ORs using the formula outlined in the “Enrichment analyses of rare substitutions” section of the Material and Methods. As WGBS data was only available for the Holstein breed, our analysis was restricted to this breed.

All analyses described in the Materials and Methods section were performed using GATK, BCFtools (version 1.21), and custom Python and R scripts.

## Results

### Detection of DNMs and rare substitutions

From the family trio segments spanning over multiple generations, we investigated a total of 378.3 Gb and 121.1 Gb of genome (cumulated length of all segments) for the Holstein and Montbéliarde breeds, respectively. In Montbéliarde, only a small fraction of this length (10.7%) was classified as callable, *i.e.*, meeting the quality criteria in all three animals of a trio to confidently detect or exclude a DNM. Due to the lack of GVCF files for several Holstein animals, the proportion of callable genome could not be estimated for this breed. A total of 3543 putative DNMs were identified, 1670 in Holstein and 1873 in Montbéliarde. Out of these DNMs, bi-allelic substitutions represented 82.3% of DNMs in Holstein and 72.0% of DNMs in Montbéliarde. Small indels (1–10 bp) accounted from 9.0% and 16.3% in Holstein and Montbéliarde, respectively. The transition-to-transversion ratios (Ti/Tv) were 2.13 for Holstein and 2.23 for Montbéliarde, with substitution proportions generally consistent between the two breeds. Regarding rare bi-allelic substitutions (*i.e.*, defined as having a MAF below 0.001 both within the breed and across all breeds in the 1000 Bull Genomes Project panel), we identified 1,802,081 such variants in theHolstein breed. The distribution of the genomic relationship coefficients of the individuals is shown in Supplementary Fig. 2. As the number of sequenced animals was smaller than 500 for the Montbéliarde breed, no rare substitution was observed at this frequency threshold. C > T transitions were markedly more frequent than T > C transitions. Moreover, their proportion among rare substitutions was higher than that observed among substitution DNMs. The Ti/Tv ratios for rare substitutions in Holstein was 2.83. Substitution proportions are shown in Table 1.

**Fig. 2:**
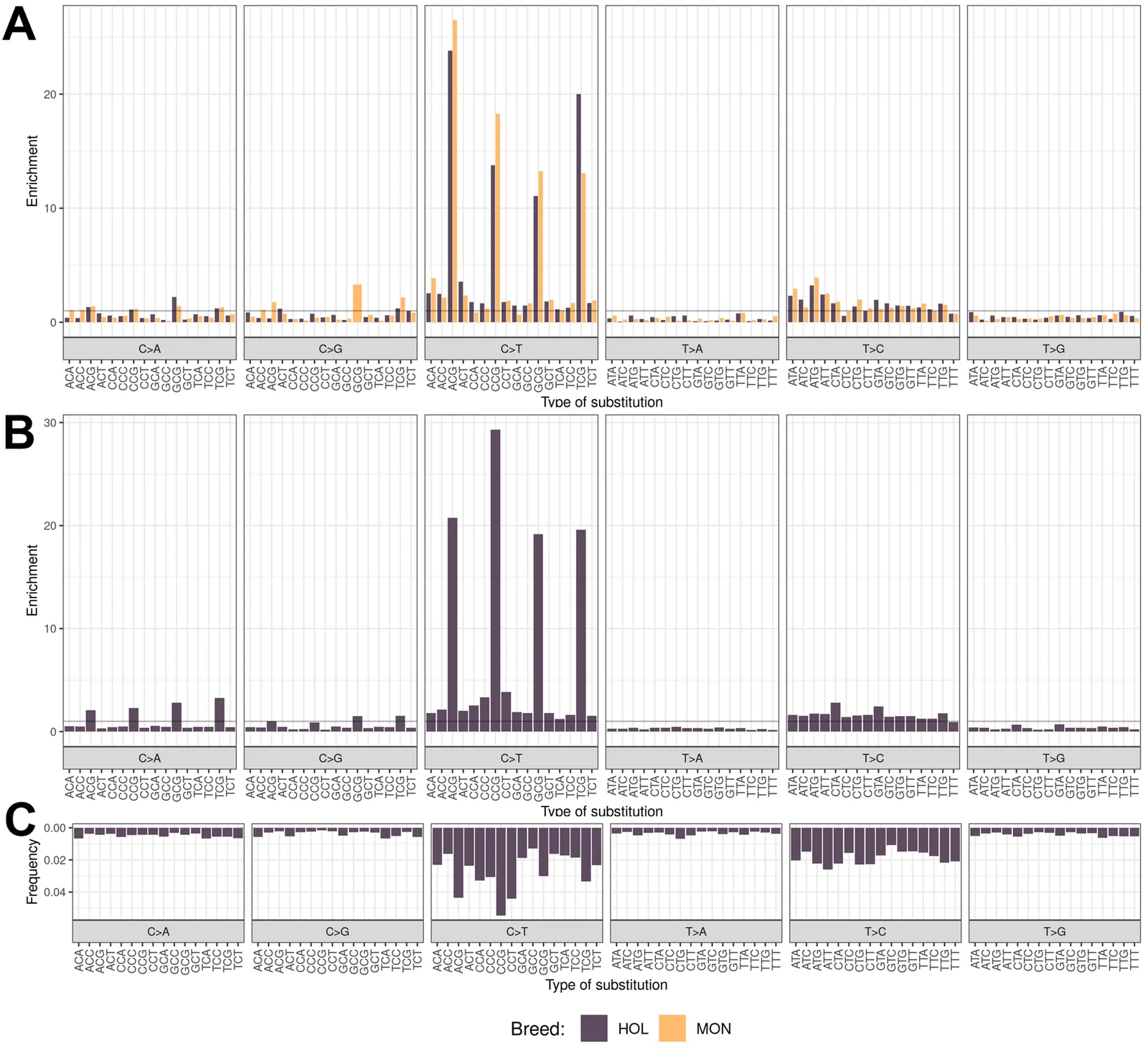
Trinucleotide contexts of substitutions **a** Enrichment of the observed substitution DNMs and **b** rare substitutions (MAF < 0.001) for each of the 96 unique substitution events (considering the trinucleotide contexts) compared to the expected balanced distribution, (considering the trinucleotide frequency observed in the reference genome). Horizontal line shows the balanced distribution (*i.e.*, 1: no enrichment nor depletion). Differences in colours represent different breeds. **c** Frequency of rare substitutions (MAF < 0.001) for each of the 96 unique substitution events

**Table 1:** Descriptive statistics of substitution DNMs and rare substitutions

	Substitution DNMs		Rare substitutions (MAF < 0.001)
	Holstein	Montbéliarde	Holstein
Total Genome (TG) explored	378.3 Gb	121.1 Gb	
Callable Genome in Gb (% of TG)	–	12.9 Gb (10.7%)	
Substitutions			
C > A	114 (7.91%)	108 (8.01%)	141,147 (7.83%)
C > G	119 (8.26%)	106 (7.86%)	104,626 (5.81%)
C > T	542 (37.61%)	498 (36.92%)	791,192 (43.90%)
T > A	88 (6.11%)	89 (6.6%)	103,046 (5.72%)
T > C	439 (30.46%)	433 (32.1%)	540,564 (30.00%)
T > G	139 (9.65%)	115 (8.52%)	121,506 (6.74%)
Total	1441	1349	1,802,081
Ti/Tv^1^	2.13	2.23	2.83

^1^Transition/transversion ratio: ([C > T] + [T > C])/([C > A] + [C > G] + [T > A] + [T > G])

### Substitution DNMs and rare substitutions are enriched for CpG > TpG transitions

Compared to the proportion of trinucleotides in the reference genome, the trinucleotide contexts of substitution DNMs and rare substitutions exhibited distinctive patterns of bias (Fig. 2, a and b). In agreement with the Ti/Tv ratios reported in Table 1, transitions (*i.e*.*,* C > T and T > C) were overrepresented (OR > 1) for most trinucleotide contexts compared to transversions, with C > T transitions showing the strongest enrichment. In addition to the general enrichment of transitions, the CpG > TpG context exhibited a markedly higher enrichment (10 < OR < 30) for both substitution DNMs and rare substitutions, and consistent OR values were observed in both breeds for substitution DNMs. Among transversions, C > A and C > G substitutions occurring in a CpG context also showed enrichment, particularly pronounced among rare substitutions, and, to a lesser extent, in substitution DNMs.

When only high-density CpG regions, *i.e.*, CpG islands (CGIs), were considered and the observed trinucleotide proportion in the reference genome of these regions was accounted for, the enrichment of CpG > TpG transitions was less pronounced (Additional file 3 Fig. S3, a and b, 2 < OR < 6).

### De novo mutation rate

The observed germline de novo mutation rate per generation was estimated at 2.05 × 10^–8^/bp in Montbéliarde. The observed de novo mutations accumulate with the number of generations separating the direct ascendants and the progeny (*P*-value = 1.8 × 10^–7^; regression weighted by the length of callable genome in each generation class) (Additional file 3 Fig. S4). As mentioned earlier, the absence of GVCF data for most Holstein animals prevented reliable estimation of the callable genome and, consequently, to compute the DNM rate for this breed.

### Substitution DNMs and rare substitutions distribution across genomic regions

**Fig. 3:**
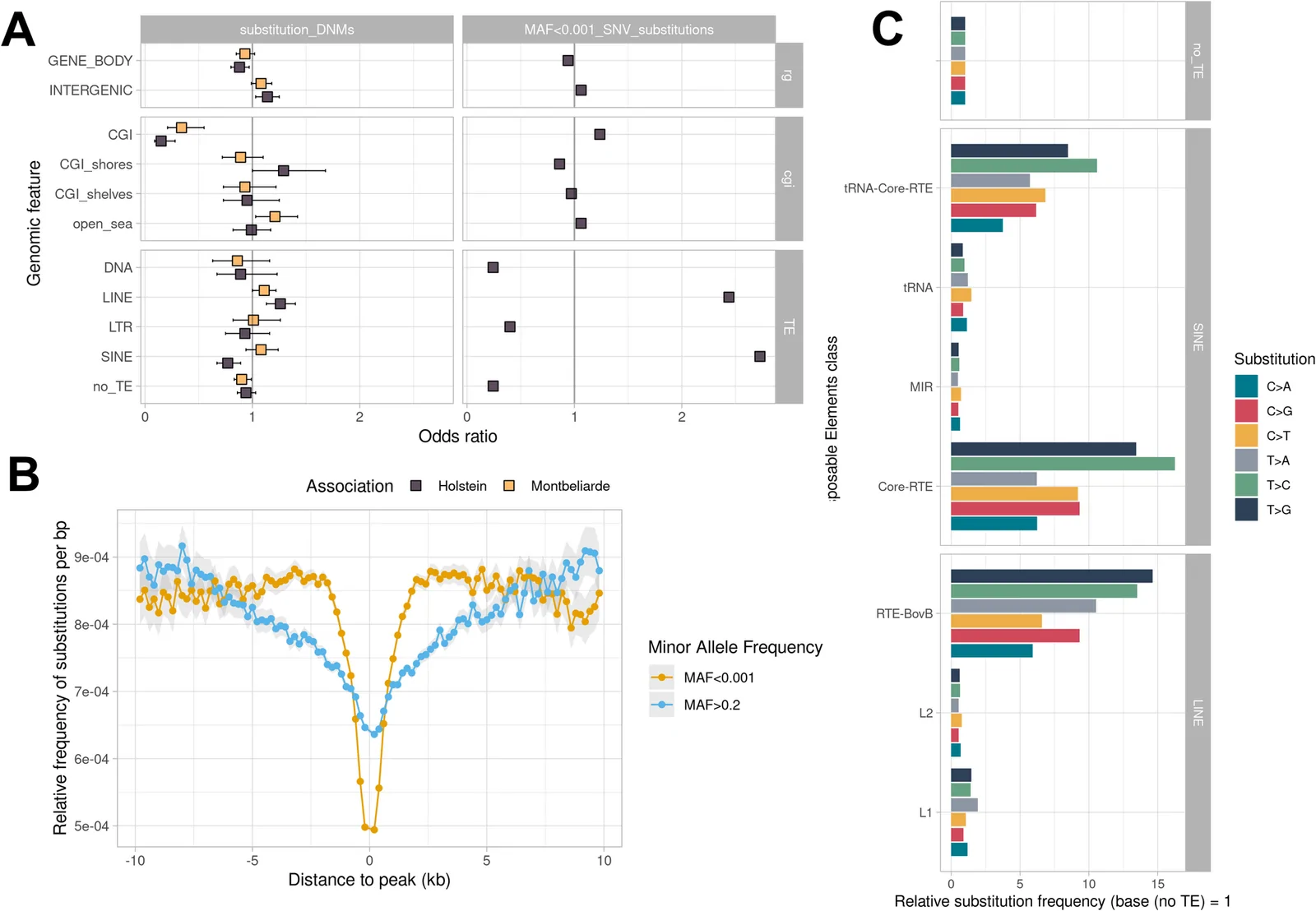
Genomic context of mutations **a** Enrichment of the observed substitution DNMs and rare substitutions (MAF < 0.001) for annotated genomic features including genomic regions (rg), CpG Island (CGI) landscape and transposable elements (TE). **b** Frequency of raresubstitutions (MAF < 0.001; yellow) and common substitutions (MAF > 0.2; blue) relative to the distance from ATAC-seq peak centres. Common substitutions were randomly selected to equal the number of rare substitutions observed. **c** Rare substitution frequency enrichment for several classes of transposable elements relative to the observed substitution rate in non-TE regions

We assessed the enrichment of substitution DNMs and rare substitutions across various genomic annotations (Fig. 3). Overall, enrichment patterns were consistent between the Holstein and Montbéliarde breeds for substitution DNMs and some similar trends were shared between substitution DNMs and rare substitutions (Fig. 3a). Rare substitutions showed a slight enrichment in intergenic regions (OR_Hol_ = 1.06). This pattern was consistent for substitution DNMs in the Holstein breed (OR_Hol_ = 1.14, CI_95%_ = [1.03–1.25]), and a similar but not significant trend was observed in the Montbéliarde breed (OR_Mon_ = 1.08, CI_95%_ = [0.99–1.18]).

Considering the DNA methylation landscape, CGI regions exhibited a significant depletion of substitution DNMs in both breeds (OR_Hol_ = 0.15; OR_Mon_ = 0.34). However, the low number of DNMs in these regions may lead to inaccuracies in OR estimates. Conversely, rare substitutions were overrepresented in CGIs (OR_Hol_ = 1.24), while the regions surrounding CGIs (CGI shores and CGI shelves) were depleted for these variants.

The transposable elements (TE) landscape also displayed patterns of both enrichment and depletion, primarily for rare substitutions. Regions that were not classified as TEs showed a tendency of lower frequency for substitution DNMs (OR_Hol_ = 0.94; OR_Mon_ = 0.91). This was more pronounced for rare substitutions (OR_Hol_ = 0.24). In contrast, both SINEs and LINEs were strongly enriched for rare substitutions (OR_Hol_ > 2.4). Specifically, several families of the SINEs and LINEs classes were highly enriched for all bi-allelic substitutions (Fig. 3c). This was especially evident for the LINE/RTE-BovB, SINE/Core-RTE and SINE/tRNA-Core-RTE families, which are considered to be evolutionarily young TEs [28].

Regarding the chromatin accessibility landscape, we observed that ATAC-seq peaks were associated with a reduced frequency of rare substitutions (Fig. 3b), starting at ~ 2.5 kb from the peaks. In contrast, the frequency of common substitutions (MAF > 0.2) declined more gradually as the distance from the peaks decreases. Nevertheless, the decreased the frequency of common variants within the ATAC-seq peaks themselves was also observed (Fig. 3b).

### CpG-to-TpG mutability is associated with local methylation level

Using previously published WGBS data [24, 25], we compared, within the Holstein breed, the proportion of CpGs with a local methylation level > 50% (considering 1000 bp bins) between all CpGs (*i.e.,* background set) and: (1) the CpG > TpG substitution DNMs or (2) the CpG > TpG rare substitutions. Results across ten tissues (including somatic tissues and gametes) are presented in Table 2. The proportion of CpGs with a local methylation level greater than 50% was higher for CpG > TpG substitution DNMs in all tissues except in vivo MII oocyte, 8-cell and 16-cell embryos. This enrichment was particularly notable for sperm, blood, liver, and adipose tissue, with *P*-values < 0.01. However, these *P*-values may be influenced by the number of CpG > TpG substitution DNMs with local methylation level available. Likewise, the proportion of CpGs with a local methylation level > 50% was higher for the rare CpG > TpG substitutions than the proportion observed for the background set (all CpGs), for all tissues. Consistent with the observations for the CpG > TpG substitution DNMs, the strongest enrichments were observed for sperm, blood, liver, and adipose tissue (OR > 3).

**Table 2:** Methylation levels for all CpGs, CpG-to-TpG substitution DNMs, and CpG-to-TpG rare substitutions (MAF < 0.001) for several tissus

	All CpGs		CpG > TpG substitution DNMs				Rare CpG > TpG substitutions			
Tissu	N	Meth > 0.5	N	Meth > 0.5	OR	*P*-value	N	Meth > 0.5	OR	95% CI
Sperm^1^	17,959,882	0.85	90	0.97	4.95	5.8e-04	215,248	0.95	3.06	[3.00–3.12]
immature oocyte^1^	9,717,989	0.39	31	0.58	2.12	0.041	86,159	0.49	1.46	[1.44–1.48]
in vivo MII oocyte^1^	6,309,251	0.42	13	0.62	2.24	0.17	47,101	0.55	1.73	[1.70–1.77]
2-cell^1^	11,091,950	0.4	36	0.61	2.37	0.016	104,217	0.49	1.44	[1.43–1.46]
4-cell^1^	10,731,037	0.34	37	0.51	2.04	0.036	98,519	0.41	1.34	[1.33–1.36]
8-cell^1^	9,748,429	0.21	32	0.25	1.26	0.52	84,758	0.26	1.34	[1.32–1.36]
16-cell^1^	15,121,571	0.29	68	0.25	0.82	0.51	167,238	0.32	1.17	[1.16–1.18]
Blood^2^	23,402,873	0.88	113	0.97	5.15	7.2e-04	270,822	0.97	5.22	[5.10–5.35]
Liver^2^	23,330,496	0.84	113	0.93	2.59	6.8e-03	270,297	0.94	3.14	[3.09–3.19]
Adipose^2^	23,401,835	0.86	113	0.99	18.38	1.8e-06	270,824	0.97	4.95	[4.84–5.05]

Results are presented only for the Holstein breed as WGBS data were available only in this breed. ^1^WGBS data obtained from NCBI Gene Expression Omnibus (GEO; http://www.ncbi.nlm.nih.gov/geo/; GSE121758), ^2^WGBS data obtained from NCBI GEO (GSE147087)

Given that the observed trinucleotide pattern of rare substitutions in CGIs differed from that observed at the whole-genome scale (Additional file 3 Fig. S3), we then focused our analysis on the local methylation level within CGI regions (Table 3). Overall, the global methylation level in CGI was found to be lower than that when all CpGs were taken into account. Nevertheless, consistent with observations from all rare CpG > TpG substitutions (Table 2), those occurring within CGIs also showed higher local methylation levels across all tissues analysed, despite the typically lower local methylation patterns observed in CGIs.

**Table 3:** Methylation levels of CpG-to-TpG rare substitutions (MAF < 0.001) in CpG islands (CGIs) for several tissues

	All CpGs in CGIs		Rare CpG > TpG substitutions in CGIs			
Tissu	N	Meth > 0.5	N	Meth > 0.5	OR	95% CI
Sperm^1^	1,651,774	0.21	3767	0.56	4.8	[4.5–5.12]
immature oocyte^1^	1,608,976	0.16	3537	0.47	4.61	[4.32–4.93]
in vivo MII oocyte^1^	1,460,480	0.14	3117	0.44	4.62	[4.3–4.96]
2-cell^1^	1,641,310	0.1	3741	0.31	3.87	[3.61–4.15]
4-cell^1^	1,637,631	0.12	3716	0.35	3.82	[3.57–4.09]
8-cell^1^	1,624,070	0.07	3674	0.21	3.33	[3.08–3.61]
16-cell^1^	1,650,965	0.11	3771	0.33	3.75	[3.51–4.02]
Blood^2^	2,306,856	0.32	4698	0.79	7.99	[7.45–8.57]
Liver^2^	2,298,199	0.29	4693	0.75	7.37	[6.9–7.88]
Adipose^2^	2,305,153	0.29	4698	0.76	7.74	[7.24–8.28]

Results are presented only for the Holstein breed as WGBS data were available only in this breed. ^1^WGBS data obtained from NCBI Gene Expression Omnibus (GEO; http://www.ncbi.nlm.nih.gov/geo/; GSE121758), ^2^WGBS data obtained from NCBI GEO (GSE147087)

## Discussion

This study investigated de novo mutations and rare substitutions in dairy cattle. A total of 3543 putative DNMs have been identified, primarily corresponding to bi-allelic substitutions. Combined with analyses of rare substitutions (MAF < 0.001) identified from a large panel of sequenced animals, our study revealed that mutations are unevenly distributed across the genome, and preferentially occur in specific trinucleotide contexts, particularly CpG sites. Specifically, CpG > TpG transitions were associated with higher local methylation levels in most tissues, and a reduced CpG > TpG mutation intensity was observed in CGIs.

Although they occur at a very low frequency, mutations can be associated with different genomic features, providing insights into fundamental biological processes [29, 30]. In line with previous studies [31, 32], substitutions showed trinucleotide context biases, mainly driven by a significant overrepresentation of CpG > TpG substitutions. It has been reported that methylation explains, at least in part, this increased mutability of C > T in CpG [8]. The CpG context was also associated with a slight overrepresentation of C > A and C > G transversions over all transversions. This mutational signature has previously been proposed as a hallmark of enzymatic demethylation [29], originating from unrepaired hydroxymethylcytosine before replication [29] and been more intense inside CpG islands. While CpGs are more prone to C > T mutations, we observed that the enrichment pattern of CpG > TpG rare mutations was markedly lower in CpG-dense regions. These findings are in agreement with previous studies in several species including Sticklebacks [33] and humans [34, 35]. This likely reflects the generally lower methylation levels observed in CpG islands [36], although Panchin et al. [37] argued that the reduced methylation levels of CpGs alone is not sufficient to explain the lower CpG > TpG mutability in CpG islands. These authors suggested that selective pressures may maintain methylated cytosine within CpG islands [37].

Our observations also revealed a significant overrepresentation of rare substitutions in several TE families, particularly those considered young and potentially active [28]. These elements are subject to substantial repression through various mechanisms, including DNA methylation [38], particularly within heterochromatic regions. In agreement with our findings, Carlson et *al.* have reported a favorable correlation between variant density and H3K9me3 [34], a histone mark associated with heterochromatin formation and transcriptional repression, implicated in the repression of transposable elements [39]. Chromatin organization has also been previously correlated with mutation rate in several species [30, 40]. Our results support these findings: rare substitutions were depleted in accessible chromatin regions (ATAC-seq peaks), more than common substitutions, which may result from purifying selection as stated in Yuan et al. [23].

The association between chromatin accessibility and a reduced regional mutation rate has been reported in previous studies [40, 41]. However, this does not imply causality; chromatin accessibility is significantly associated with replication timing (RT), which has been connected to mutation rate [30, 42]. Behind this overall mutation rate, distinct patterns in the relative substitution rates were observed in relation to different chromatin characteristics or histone marks [34, 40]. Unexpectedly, our observations from the rare substitution frequency in ATAC-seq peaks differ from previous work conducted in the same breed [23]. This discrepancy may be partly explained by differences in the subset of variants used in our analysis, which could influence the distribution of rare alleles across functional regions. Therefore, the results presented here should be interpreted within the context of the specific filtering criteria and analytical conditions adopted in this study.

The Ti/Tv ratio observed for substitution DNMs was consistent with previous findings for cattle, based on family trios [5], or WGS observations [15], and contributed to the enrichment of DNMs across different trinucleotide contexts. The higher Ti/Tv ratio for rare substitutions (2.83) may be the result of a bias when selecting very rare variants, especially when filtering against the 1000 Bull Genome Project, as Holstein contributed to a significant proportion of the panel.

Harland et al. [31] estimated that 30–50% of DNMs in gamete arise from early cleavage cell divisions, resulting in frequent mosaicism. Here, our focus was primarily on C > T mutations in CpG contexts. Although we observed an association between local methylation levels and C > T substitutions, the early stages of development did not display the strongest associations. As our study was based on several generations, we could not directly assess mosaicism, as such mutations would either have been transmitted or lost by the sampled generation.

Mutation studies commonly employ two main approaches: analysis of de novo mutations identified through trio pedigreesequencing and investigation of rare variants (*i.e.*, those with low allelic frequencies in the population). Trio-based approaches are robust, and several designs enable to trace the parental origin of DNMs and estimate the proportion of mosaic DNMs. However, it requires the analysis of multiple trios to quantitatively describe mutational patterns. The latter approach requires numerous sequences to identify rare variants and is more limited in the characterization of mutations (*e.g.*, tracing of the parental origin). Nevertheless, it facilitates the identification of a substantial number of variants that are relatively unaffected by evolutionary or selective forces [34] and enables certain mutational processes to be distinguished and mutagenic effects to be characterized [29, 34, 35, 43]. This approach enabled us to conduct more detailed studies of CpG > TpG mutations in CpG islands, as well as other investigations, compared to the more limited dataset of DNMs obtained from family trios. Our first design was a complementary approach to the trio pedigree sequencing. We relied on pedigree trio spanning over generations to maximize the number of generations between progeny and ancestors in order to recover an “accumulation” of de novo mutations. This indirect approach still allowed us to compute a germline mutation rate per generation. However, it relies on existing data for which the first goal was not to detect DNMs—WGS was in average of limited quality compared to other pedigree-based studies—which introduce uncertainties and strongly impact the size of informative genome (*i.e.*, callable genome), much lower than our initial expectations. Although computational estimates of shared DNA between a progeny and all direct ascendants with available WGS sometimes exceeded 80%, the proportion of usable data was an order of magnitude lower. Also, this approach relied on IBD detection, which is not perfect and introduces uncertainty, particularly around the breakpoints of the segments. Lack of stringent filtering could result in clusters of DNMs in close proximity on the same IBD segment. This is extremely unlikely under realistic mutation rates and would be more likely to reflect technical artefacts. For this reason, we applied strict filters to minimise false positives, even if this excluded a small number of true DNMs.

The apparent per generation germline mutation rate was higher than in previous reports [5]. Gene conversion [44] is a plausible mechanism that could explain the elevated mutation rate observed in our study. We identify de novo mutations on haplotypes in descendants that are inferred to be IBD with those of an ancestor separated by several generations. However, in an intermediate generation, a short tract within an otherwise IBD segment may undergo gene conversion. This non-reciprocal event copies alleles from the homologous chromosome onto the transmitted haplotype. As a result, alleles that were already present on the homologous chromosome may be introduced into the studied haplotype and subsequently misidentified as de novo mutations in the descendant under our study design. Of note, although we applied stringent filtering, we cannot exclude the possibility that some detected DNMs were false positives, as validating DNMs using a robust trio-sequencing approach can result in a significant proportion being eliminated [19].

De novo mutations are more likely to be deleterious than inherited variants because they did not yet experience counter-selection [45]. However, our study design presents two biases that limit the discovery of deleterious mutations: (1) animals (both ancestors and progeny) are mostly fertile breeding animals, without apparent fitness problem, and (2) we consider pedigree trios spanning several generations, which enables the potential counter-selection of deleterious or unfavourable de novo mutations to be eliminated in intermediate generations.

## Conclusions

We studied rare variants in dairy cattle obtained from (1) trios spanning multiple generations and (2) large-scale genomic resources. In light of the recent genomic annotation data, we are primarily confirming observations that have been made in other species. Although this provides interesting insights, crucial data for a more in-depth investigation of mutations in cattle is still missing, such as replication timing maps, to improve the resolution of observed mechanisms and better describe possible redundancies between information sources.

## Supplementary Information

Below is the link to the electronic supplementary material.

Supplementary Material 1

Supplementary Material 2

## Data Availability

Raw sequencing data used in this study were obtained from 1000 bull Genomes Project [10, 46] and from other projects (accession numbers PRJEB64022 and PRJEB64023) available at European Nucleotide Archive (ENA, https://www.ebi.ac.uk/ena). Genotypes are commercial information belonging to the French breeders and are not publicly available. The list of DNMs and rare substitutions are available as Supplementary Tables.

## References

[1] Lynch M, Ackerman MS, Gout JF, Long H, Sung W, Thomas WK, et al. Genetic drift, selection and the evolution of the mutation rate. Nat Rev Genet. 2016;17:704–14. 27739533 10.1038/nrg.2016.104

[2] Bourneuf E, Otz P, Pausch H, Jagannathan V, Michot P, Grohs C, et al. Rapid discovery of de novo deleterious mutations in cattle enhances the value of livestock as model species. Sci Rep. 2017;7:11466. 28904385 10.1038/s41598-017-11523-3 PMC5597596

[3] Corbeau J, Grohs C, Jourdain J, Boussaha M, Besnard F, Barbat A, et al. A recurrent de novo missense mutation in COL1A1 causes osteogenesis imperfecta type II and preterm delivery in Normande cattle. Genet Sel Evol. 2024;56:39. 38773368 10.1186/s12711-024-00909-3 PMC11107018

[4] Bergeron LA, Besenbacher S, Zheng J, Li P, Bertelsen MF, Quintard B, et al. Evolution of the germline mutation rate across vertebrates. Nature. 2023;615:285–91. 36859541 10.1038/s41586-023-05752-y PMC9995274

[5] Harland C, Durkin K, Artesi M, Karim L, Cambisano N, Deckers M, et al. Rate of de novo mutation in dairy cattle and potential impact of reproductive technologies. In: Proceedings of the World Congress on Genetics Applied to Livestock Production: 11–16 February 2018. Auckland, 2018.

[6] Kong A, Frigge ML, Masson G, Besenbacher S, Sulem P, Magnusson G, et al. Rate of de novo mutations and the importance of father’s age to disease risk. Nature. 2012;488:471–5. 22914163 10.1038/nature11396 PMC3548427

[7] Conrad DF, Keebler JEM, DePristo MA, Lindsay SJ, Zhang Y, Casals F, et al. Variation in genome-wide mutation rates within and between human families. Nat Genet. 2011;43:712–4. 21666693 10.1038/ng.862 PMC3322360

[8] Walser JC, Furano AV. The mutational spectrum of non-CpG DNA varies with CpG content. Genome Res. 2010;20:875–82. 20498119 10.1101/gr.103283.109 PMC2892088

[9] Lee YL, Bouwman AC, Harland C, Bosse M, Moreira GCM, Veerkamp RF, et al. The rate of de novo structural variation is increased in in vitro–produced offspring and preferentially affects the paternal genome. Genome Res. 2023;33:1455–64. 37793781 10.1101/gr.277884.123 PMC10620045

[10] Daetwyler HD, Capitan A, Pausch H, Stothard P, Van Binsbergen R, Brøndum RF, et al. Whole-genome sequencing of 234 bulls facilitates mapping of monogenic and complex traits in cattle. Nat Genet. 2014;46:858–65. 25017103 10.1038/ng.3034

[11] Suin A, Marcuzzo C, Eché C, Dréau A, Birbes C, Di Franco A, et al. Major contribution to the bovine pangenome: whole genome sequences, SNPs, and structural variants of 154 bulls from 14 breeds. In: Proceedings of the Plant and Animal Genome Conference (PAG 31): 12–17 January 2024; San Diego. 2024.

[12] IDELE. VARUME. 2024. https://idele.fr/detail-dossier/varume-resultats-2024. Accessed 17 Apr 2025

[13] Li H, Durbin R. Fast and accurate short read alignment with Burrows-Wheeler transform. Bioinformatics. 2009;25:1754–60. 19451168 10.1093/bioinformatics/btp324 PMC2705234

[14] McKenna A, Hanna M, Banks E, Sivachenko A, Cibulskis K, Kernytsky A, et al. The Genome analysis toolkit: a MapReduce framework for analyzing next-generation DNA sequencing data. Genome Res. 2010;20:1297–303. 20644199 10.1101/gr.107524.110 PMC2928508

[15] Boussaha M, Michot P, Letaief R, Hozé C, Fritz S, Grohs C, et al. Construction of a large collection of small genome variations in French dairy and beef breeds using whole-genome sequences. Genet Sel Evol. 2016;48:87. 27846802 10.1186/s12711-016-0268-z PMC5111192

[16] Jourdain J, Capitan A, Saintilan R, Hozé C, Fouéré C, Fritz S, et al. Genetic parameters, genome-wide association study, and selection perspective on gestation length in 16 French cattle breeds. J Dairy Sci. 2024;107:8157–69. 39004135 10.3168/jds.2023-24632

[17] Sargolzaei M, Chesnais JP, Schenkel FS. A new approach for efficient genotype imputation using information from relatives. BMC Genomics. 2014;15:478. 24935670 10.1186/1471-2164-15-478 PMC4076979

[18] Liang X, Yang S, Wang D, Knief U. Characterization and distribution of de novo mutations in the zebra finch. Commun Biol. 2024;7:1–13. 39358581 10.1038/s42003-024-06945-5 PMC11447093

[19] Rochus CM, Steensma MJ, Bink MCAM, Huisman AE, Harlizius B, Derks MFL, et al. Estimating mutation rate and characterising single nucleotide de novo mutations in pigs. Genet Sel Evol. 2025;57:21. 40229661 10.1186/s12711-025-00967-1 PMC11995543

[20] Purcell S, Neale B, Todd-Brown K, Thomas L, Ferreira MAR, Bender D, et al. PLINK: a tool set for whole-genome association and population-based linkage analyses. Am J Hum Genet. 2007;81:559–75. 17701901 10.1086/519795 PMC1950838

[21] Harland C. Germline mutations in Bos taurus. PhD thesis; Université de Liège. 2018.

[22] UCSC Genome Browser Gateway. 2024. https://genome-euro.ucsc.edu/cgi-bin/hgGateway. Accessed 15 Apr 2026.

[23] Yuan C, Tang L, Lopdell T, Petrov VA, Oget-Ebrad C, Moreira GCM, et al. An organism-wide ATAC-seq peak catalog for the bovine and its use to identify regulatory variants. Genome Res. 2023;33:1848–64. 37751945 10.1101/gr.277947.123 PMC10691486

[24] Duan JE, Jiang ZC, Alqahtani F, Mandoiu I, Dong H, Zheng X, et al. Methylome dynamics of bovine gametes and in vivo early embryos. Front Genet. 2019. 10.3389/fgene.2019.00512. 31191619 10.3389/fgene.2019.00512 PMC6546829

[25] Zhou Y, Liu S, Hu Y, Fang L, Gao Y, Xia H, et al. Comparative whole genome DNA methylation profiling across cattle tissues reveals global and tissue-specific methylation patterns. BMC Biol. 2020;18:85. 32631327 10.1186/s12915-020-00793-5 PMC7339546

[26] Kohailan M, Aamer W, Syed N, Padmajeya S, Hussein S, Sayed A, et al. Patterns and distribution of de novo mutations in multiplex Middle Eastern families. J Hum Genet. 2022;67:579–88. 35718832 10.1038/s10038-022-01054-9 PMC9510050

[27] Smith ZD, Hetzel S, Meissner A. DNA methylation in mammalian development and disease. Nat Rev Genet. 2025;26:7–30. 39134824 10.1038/s41576-024-00760-8

[28] Wang C, Lei B, Bao Y, Wang Z, Chen C, Zhang Y, et al. Multi-omics analysis reveals critical cis-regulatory roles of transposable elements in livestock genomes. iScience. 2025;28:112049. 40104067 10.1016/j.isci.2025.112049 PMC11914811

[29] Seplyarskiy VB, Soldatov RA, Koch E, McGinty RJ, Goldmann JM, Hernandez RD, et al. Population sequencing data reveal a compendium of mutational processes in the human germ line. Science. 2021;373:1030–5. 34385354 10.1126/science.aba7408 PMC9217108

[30] Seplyarskiy VB, Sunyaev S. The origin of human mutation in light of genomic data. Nat Rev Genet. 2021;22:672–86. 34163020 10.1038/s41576-021-00376-2

[31] Harland C, Charlier C, Karim L, Cambisano N, Deckers M, Mni M, et al. Frequency of mosaicism points towards mutation-prone early cleavage cell divisions in cattle. bioRxiv. 2016; 10.1101/079863

[32] Shojaeisaadi H, Schoenrock A, Meier MJ, Williams A, Norris JM, Palmer ND, et al. Mutational signature analyses in multi-child families reveal sources of age-related increases in human germline mutations. Commun Biol. 2024;7:1451. 39506086 10.1038/s42003-024-07140-2 PMC11541588

[33] Zhang C, Reid K, Sands AF, Fraimout A, Schierup MH, Merilä J. De novo mutation rates in sticklebacks. Mol Biol Evol. 2023. 10.1093/molbev/msad192. 37648662 10.1093/molbev/msad192 PMC10503787

[34] Carlson J, Locke AE, Flickinger M, Zawistowski M, Levy S, Myers RM, et al. Extremely rare variants reveal patterns of germline mutation rate heterogeneity in humans. Nat Commun. 2018;9:3753. 30218074 10.1038/s41467-018-05936-5 PMC6138700

[35] Youk J, An Y, Park S, Lee JK, Ju YS. The genome-wide landscape of C:G > T:A polymorphism at the CpG contexts in the human population. BMC Genomics. 2020;21:270. 10.1186/s12864-020-6674-1 PMC7106825 32228436

[36] Illingworth RS, Gruenewald-Schneider U, Webb S, Kerr ARW, James KD, Turner DJ, et al. Orphan CpG islands identify numerous conserved promoters in the mammalian genome. PLoS Genet. 2010;6:e1001134. 20885785 10.1371/journal.pgen.1001134 PMC2944787

[37] Panchin AY, Makeev VJ, Medvedeva YA. Preservation of methylated CpG dinucleotides in human CpG islands. Biol Direct. 2016;11:11. 27005429 10.1186/s13062-016-0113-x PMC4804638

[38] Li C, Luscombe NM. Nucleosome positioning stability is a modulator of germline mutation rate variation across the human genome. Nat Commun. 2020;11:1363. 32170069 10.1038/s41467-020-15185-0 PMC7070026

[39] Gruhn WH, Tang WWC, Dietmann S, Alves-Lopes JP, Penfold CA, Wong FCK, et al. Epigenetic resetting in the human germ line entails histone modification remodeling. Sci Adv. 2023;9:eade1257. 36652508 10.1126/sciadv.ade1257 PMC9848478

[40] Makova KD, Hardison RC. The effects of chromatin organization on variation in mutation rates in the genome. Nat Rev Genet. 2015;16:213–23. 25732611 10.1038/nrg3890 PMC4500049

[41] Supek F, Lehner B. Scales and mechanisms of somatic mutation rate variation across the human genome. DNA Repair. 2019;81:102647. 31307927 10.1016/j.dnarep.2019.102647

[42] Yehuda Y, Blumenfeld B, Mayorek N, Makedonski K, Vardi O, Cohen-Daniel L, et al. Germline DNA replication timing shapes mammalian genome composition. Nucleic Acids Res. 2018;46:8299–310. 29986092 10.1093/nar/gky610 PMC6144785

[43] Zhou Y, He F, Pu W, Gu X, Wang J, Su Z. The impact of DNA methylation dynamics on the mutation rate during human germline development. G3 Bethesda. 2020;10:3337–46. 32727923 10.1534/g3.120.401511 PMC7466984

[44] Chen JM, Cooper DN, Chuzhanova N, Férec C, Patrinos GP. Gene conversion: mechanisms, evolution and human disease. Nat Rev Genet. 2007;8:762–75. 17846636 10.1038/nrg2193

[45] Veltman JA, Brunner HG. De novo mutations in human genetic disease. Nat Rev Genet. 2012;13:565–75. 22805709 10.1038/nrg3241

[46] Hayes BJ, Daetwyler HD. 1000 bull genomes project to map simple and complex genetic traits in cattle: pplications and outcomes. Annu Rev Anim Biosci. 2019;7:89–102. 30508490 10.1146/annurev-animal-020518-115024

