# Supplementary material for: Effect of methylation on genome mutability in cattle
Source: Genet Sel Evol. 2026 Jun 17;58:42. doi: 10.1186/s12711-026-01051-y (PMC13491744; doi:10.1186/s12711-026-01051-y)
Supplement: Supplementary file 1 [file 12711_2026_1051_MOESM2_ESM.pdf]

Additional file 3: Figures S1 to S4

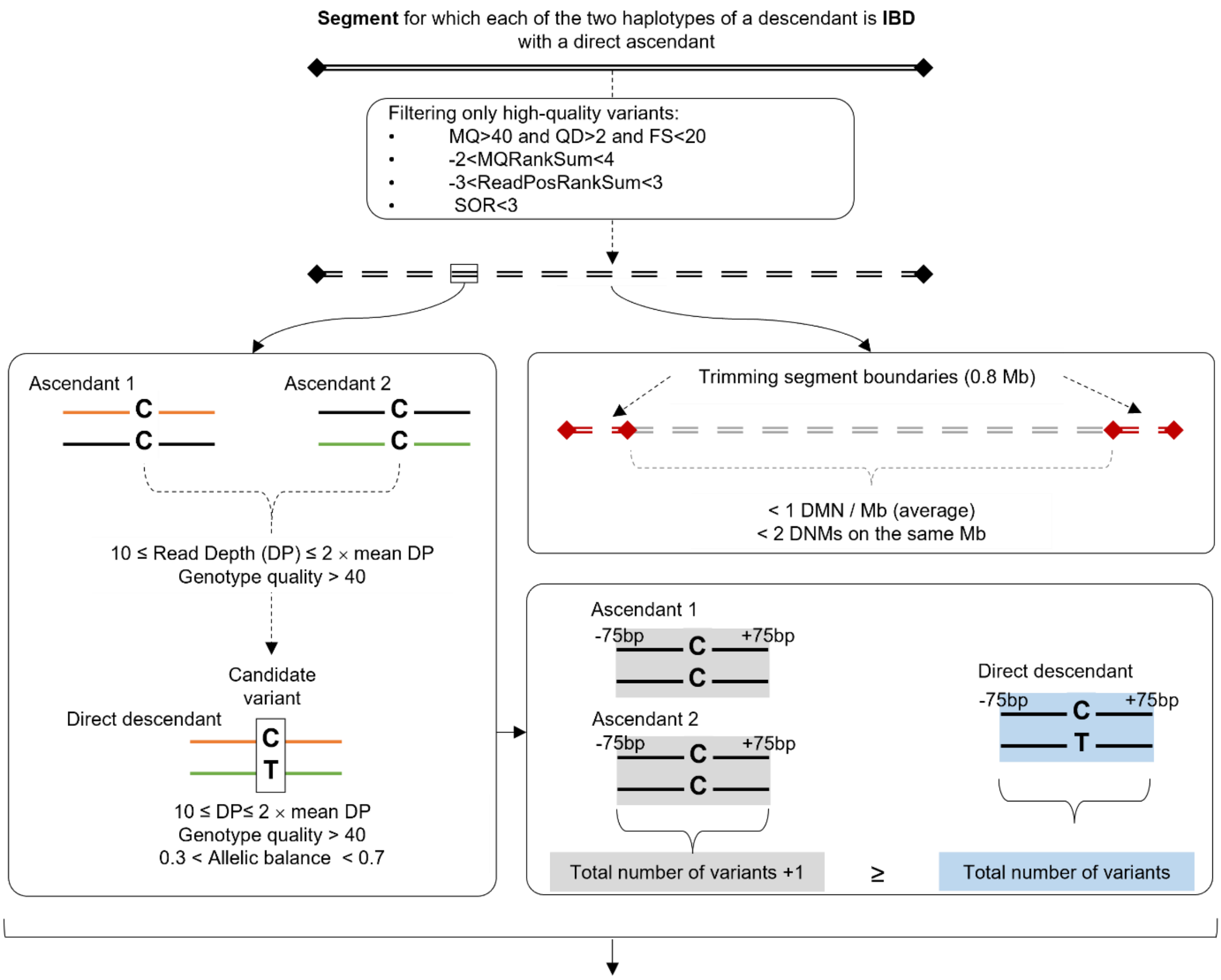

Putative DNM

Figure S1. Main steps of *de novo* mutation (DNM) filtering. From a family trio segment, corresponding to a genomic region for which each of the two haplotypes (orange and green) of a progeny is shared with a direct ascendant and considered to be inherited from that ascendant (Identical-by-Descent, IBD), stringent filtering is applied to variants. From the remaining variants, the candidate DNM undergoes a second set of filtering based on read depth, genotype quality and allelic balance. Then, nearby region (+/-75bp) in which the candidate DNM is located is examined for other variants in the area that could indicate ascendant-descendant incompatibility. In the family trio segment, reduced on each side by 0.8 Mb due to inaccuracies around the exact boundaries, the number of candidates DNMs in the region is examined. Only candidates DNMs in a region with very few DNMs observed (statistics based (1) on the reduced segment and (2) within the Mb of the candidate DNM) are retained.

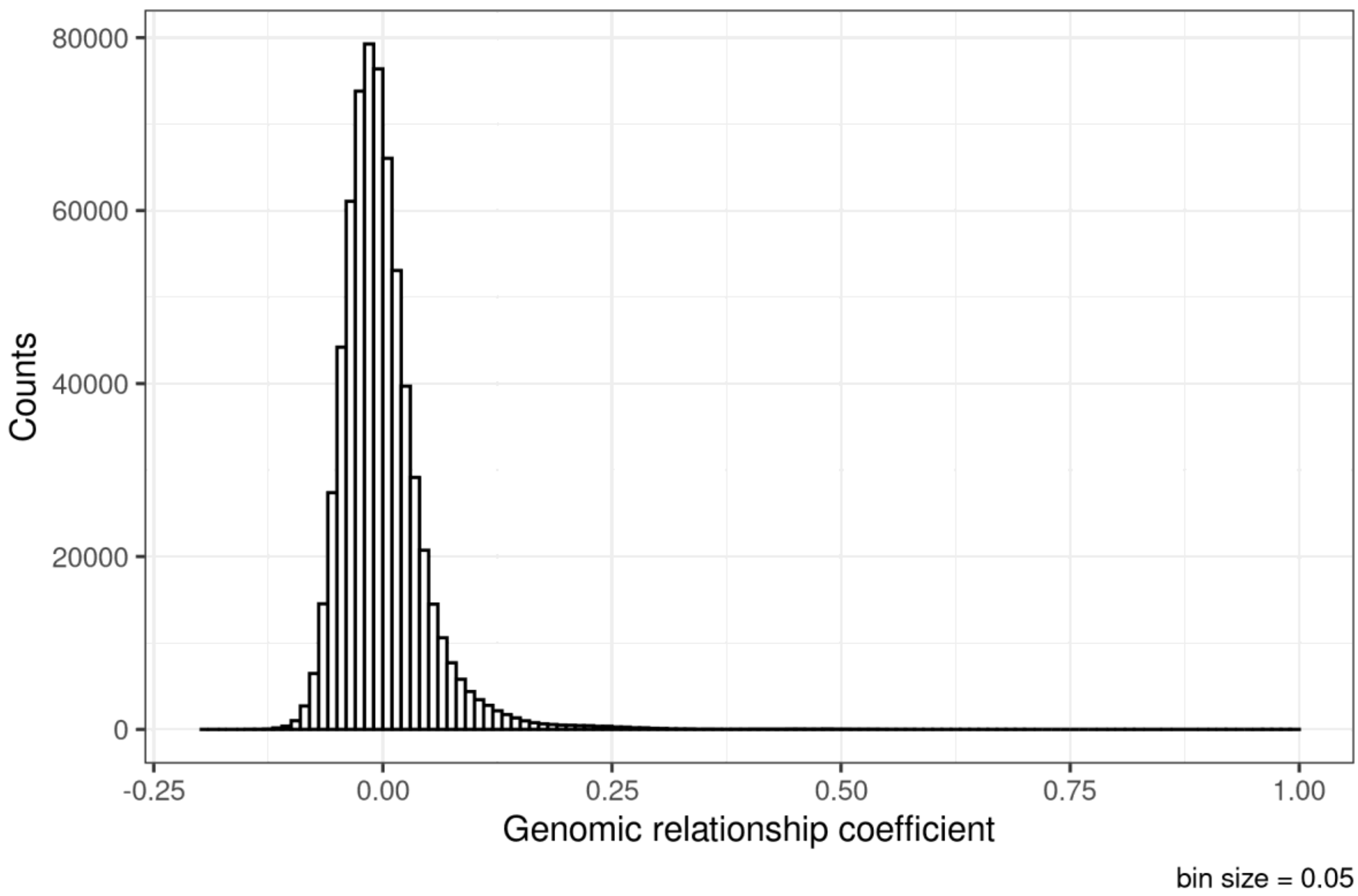

Figure S2. Distribution of the genomic relationship coefficients. The coefficients are obtained from the genomic relationship matrix computed for 50K genotypes of the 1,148 sequenced Holstein animals.

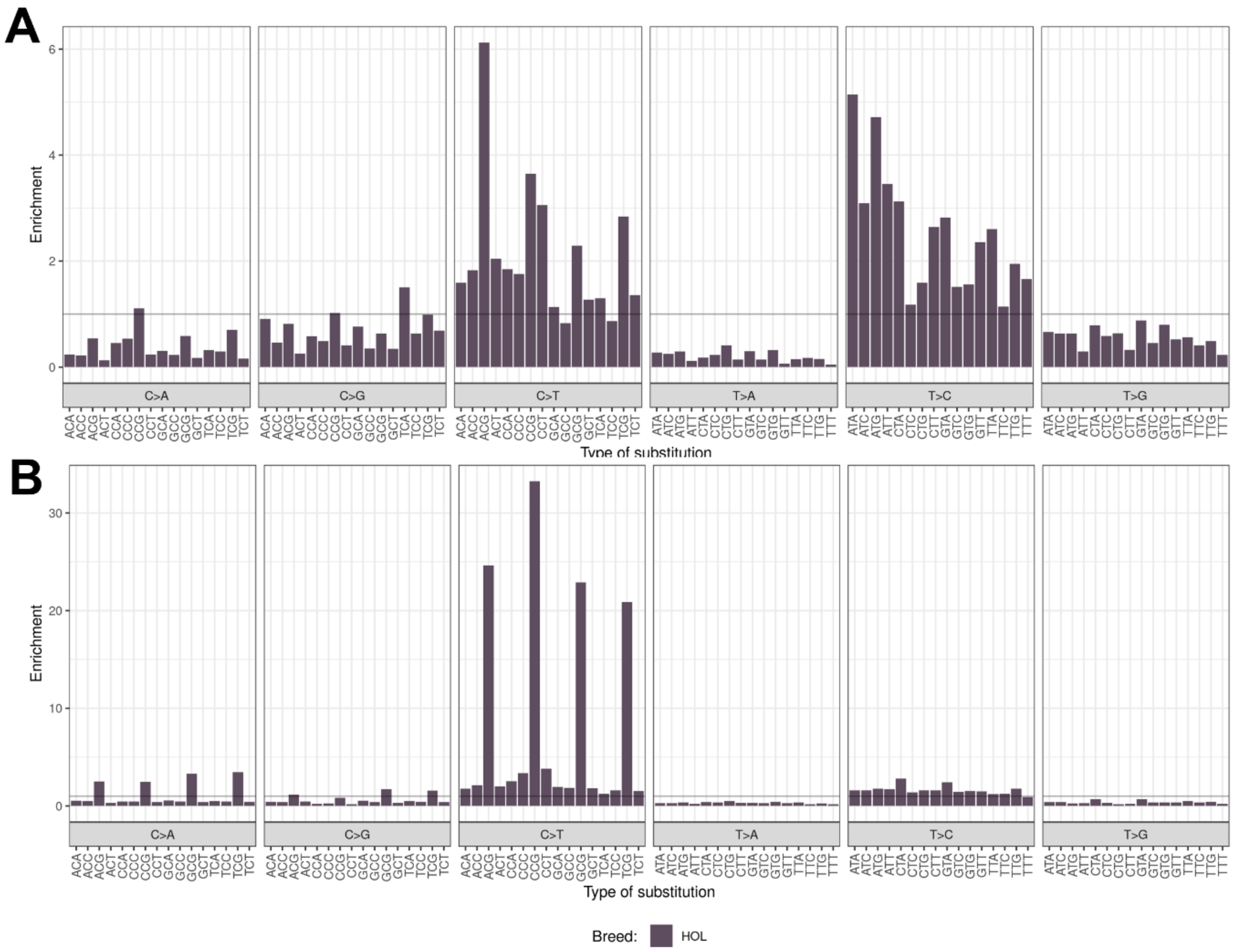

Figure S3. Rare substitutions and CpG islands. (a) Enrichment of the rare substitutions inside or (b) outside CpG islands for each of the 96 unique substitution events (considering the trinucleotide contexts) compared to the expected balanced distribution, (considering the trinucleotide frequency observed in the reference genome for the genomic region considered). Horizontal line shows the balanced distribution (i.e., 1: no enrichment nor depletion).

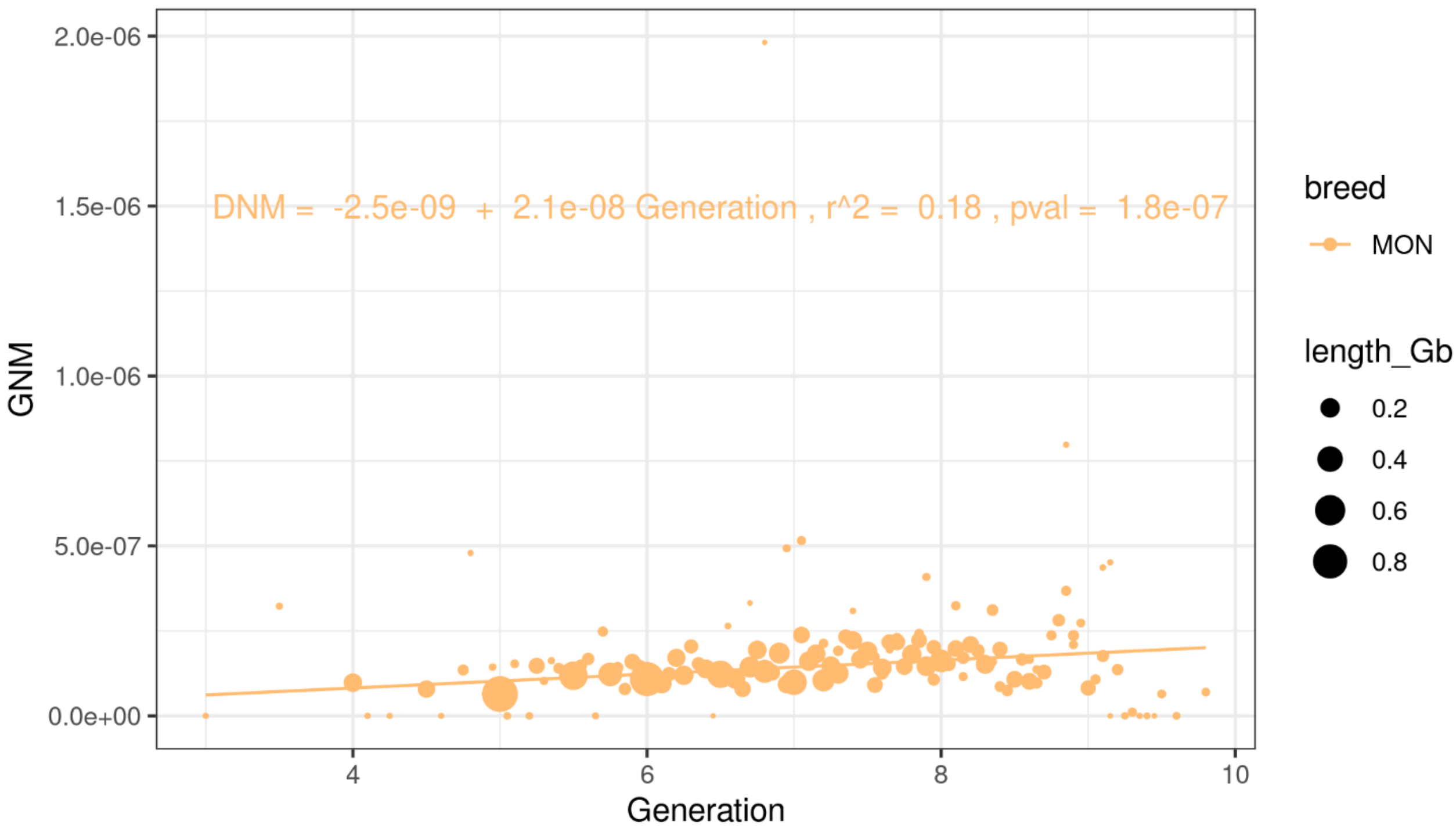

Figure S4. Mutation rate per generation in the Montbéliarde breed. Mutation rate per generation, corresponding to the sum of DNMs identified for a generation class divided by the length of callable genome investigated for the corresponding generation class. The yellow line represents the expected mutation rate per class (Mutation rate per generation × generation; obtained from the regression of the mutation rate in each generation class, weighted by the callable genome length in that class).
